# Single-Cell RNA-Seq Uncovers Robust Glial Cell Transcriptional Changes in Methamphetamine-Administered Mice

**DOI:** 10.3390/ijms26020649

**Published:** 2025-01-14

**Authors:** Abiola Oladapo, Uma Maheswari Deshetty, Shannon Callen, Shilpa Buch, Palsamy Periyasamy

**Affiliations:** Department of Pharmacology and Experimental Neuroscience, University of Nebraska Medical Center, Omaha, NE 68198, USA; abiola.oladapo@unmc.edu (A.O.); udeshetty@unmc.edu (U.M.D.); scallen@unmc.edu (S.C.); sbuch@unmc.edu (S.B.)

**Keywords:** astrocytes, methamphetamine, microglia, scRNA sequencing, oligodendrocytes

## Abstract

Methamphetamine is a highly addictive stimulant known to cause neurotoxicity, cognitive deficits, and immune dysregulation in the brain. Despite significant research, the molecular mechanisms driving methamphetamine-induced neurotoxicity and glial cell dysfunction remain poorly understood. This study investigates how methamphetamine disrupts glial cell function and contributes to neurodevelopmental and neurodegenerative processes. Using single-cell RNA sequencing (scRNA-seq), we analyzed the transcriptomes of 4000 glial cell-associated genes from the cortical regions of mice chronically administered methamphetamine. Methamphetamine exposure altered the key pathways in astrocytes, including the circadian rhythm and cAMP signaling; in microglia, affecting autophagy, ubiquitin-mediated proteolysis, and mitophagy; and in oligodendrocytes, disrupting lysosomal function, cytoskeletal regulation, and protein processing. Notably, several transcription factors, such as *Zbtb16*, *Hif3a*, *Foxo1*, and *Klf9*, were significantly dysregulated in the glial cells. These findings reveal profound methamphetamine-induced changes in the glial transcriptomes, particularly in the cortical regions, highlighting potential molecular pathways and transcription factors as targets for therapeutic intervention. This study provides novel insights into the glial-mediated mechanisms of methamphetamine toxicity, contributing to our understanding of its effects on the central nervous system and laying the groundwork for future strategies to mitigate its neurotoxic consequences.

## 1. Introduction

Amidst the ongoing stimulant crisis, methamphetamine has resurfaced as a significant public health challenge due to its increased production, illegal distribution, and consumption in recent years [1,2]. The 2020 United Nations World Drug Report estimates that globally, approximately 27 million individuals, representing 0.5% of the adult population, engage in the use of amphetamine-type stimulants, including methamphetamine [3,4]. In the United States, methamphetamine use surged by an estimated 40% between 2016 and 2018, with a subsequent rise between 2018 and 2019 [3,4]. A recent study involving one million patients in the United States reported a staggering 486.7% increase in methamphetamine-positive urine tests between 2013 and 2019 [5].

Methamphetamine is a potent psychostimulant that acts on the central nervous system (CNS), producing euphoria and a heightened sense of well-being. Its intense reinforcing properties make it highly susceptible to abuse, leading to methamphetamine use disorder (MUD) [6]. Methamphetamine primarily functions by promoting the release of neurotransmitters, including dopamine, serotonin, noradrenaline, and adrenaline, from nerve terminals in the CNS and the peripheral nervous system through distinct mechanistic pathways [7]. Over the last decade, there has been an immense growth in our understanding of methamphetamine-mediated molecular pathways, implicating neurotoxicity, excitotoxicity, oxidative stress, and neuroinflammation [8].

Methamphetamine-induced neuroinflammation has been closely linked to the exaggerated activation of astrocytes and microglia, resulting in the overproduction and accumulation of neurotoxic molecules [9]. Additionally, methamphetamine is known to disrupt mitochondrial homeostasis in neurons and microglia, further contributing to oxidative stress and neurodegeneration [10]. It has also been demonstrated that methamphetamine mediates neuroinflammation, partly through its direct binding to toll-like receptor 4 (TLR4) in the ventral tegmental area, leading to an elevated surge of dopamine in the nucleus accumbens shell [11]. Methamphetamine also facilitates neuroinflammation via NLRP3 inflammasome signaling by upregulating caspase-1 and ASC aggregation, ultimately resulting in the secretion of interleukin-1β, which drives microglia-mediated neurotoxicity [12].

Methamphetamine-induced cellular dysregulation in neurons and glial cells impairs neural processing, alters reward motivation, and reduces prefrontal cortical control, potentially progressing to neurodegenerative impairments [13,14,15,16]. These combined effects could likely facilitate the onset and maintenance of substance use behavior [2,17]. In this context, glial cells—microglia, astrocytes, and oligodendrocytes—play an active role in interacting with neurons and influencing methamphetamine-associated neuropathogenesis [10,18]. Given the substantial impact of methamphetamine on the major CNS cell types, it is essential to investigate the methamphetamine-mediated signaling pathways at the cellular and molecular levels. Single-cell RNA sequencing (scRNA-seq) has emerged as a transformative tool for identifying distinct brain cell subtypes and characterizing the transcriptional changes in neurodegenerative and neuropsychiatric disorders [19]. This technology has also been employed to study the transcriptional responses of brain cell types to substances, such as morphine, cocaine, and alcohol [20,21,22]. In this study, we utilized scRNA-seq to explore the cell type-specific effects of methamphetamine on mouse brains, with the goal of identifying the potential key signaling pathways involved in methamphetamine-associated CNS changes.

## 2. Results

### 2.1. Identification of Distinct Cell Types in the Mouse Cortexes via Unbiased scRNA-Seq

**Figure 1:**
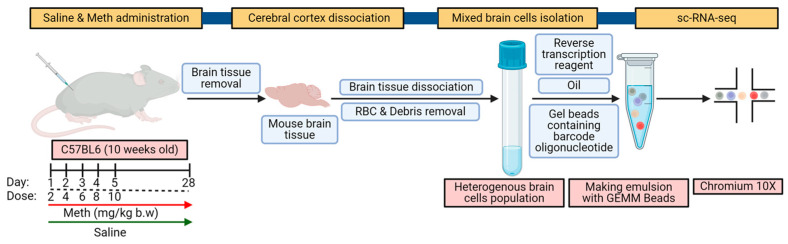
Schematic of experimental workflow for scRNA sequencing (credit: BioRender.com).

Herein, we examined cell heterogeneity and methamphetamine-mediated changes in gene expression profiles in wild-type mice. We analyzed mRNA transcripts from single cells isolated from the cortical brain regions of both saline- and methamphetamine-administered mice using the 10x Genomics platform (Figure 1).

**Figure 2:**
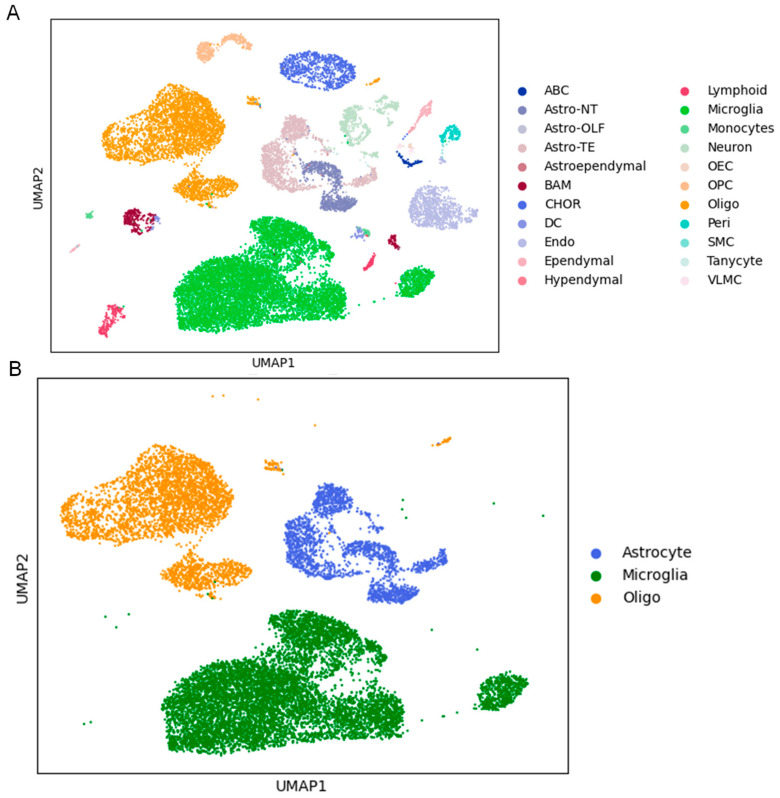
Single-cell gene expression analysis of cortical brain regions in saline- and Meth-administered wild-type mice. (**A**) UMAP displays distinct cell types in both saline and Meth groups. (**B**) UMAP displays specific glial cell types, astrocytes, microglia and oligodendrocytes, in both saline and Meth groups.

Cortical brain regions were dissected after 28 days of saline or chronic methamphetamine exposure. The raw sequencing data were processed using the 10x Genomics Cell Ranger 2.0.0 pipeline, followed by quality control. Analysis yielded 28,311 high-quality cells, representing 24 distinct cell types (Figure 2A) identified using MapMyCells, with the top 4000 genes selected using the Wilcoxon rank-sum test. The identified glial cell clusters included astrocytes, microglia, and oligodendrocytes (Figure 2B). A significant reduction in cell number was observed across multiple cell types in the cortical regions of the methamphetamine-administered mice compared to that of the saline group (Figure S1). Next, we identified the top 25 upregulated and downregulated (Figure S2A–F) DEGs in the glial cell populations.

### 2.2. Dysregulated Molecular Pathways in Astrocytes After Meth Administration

**Figure 3:**
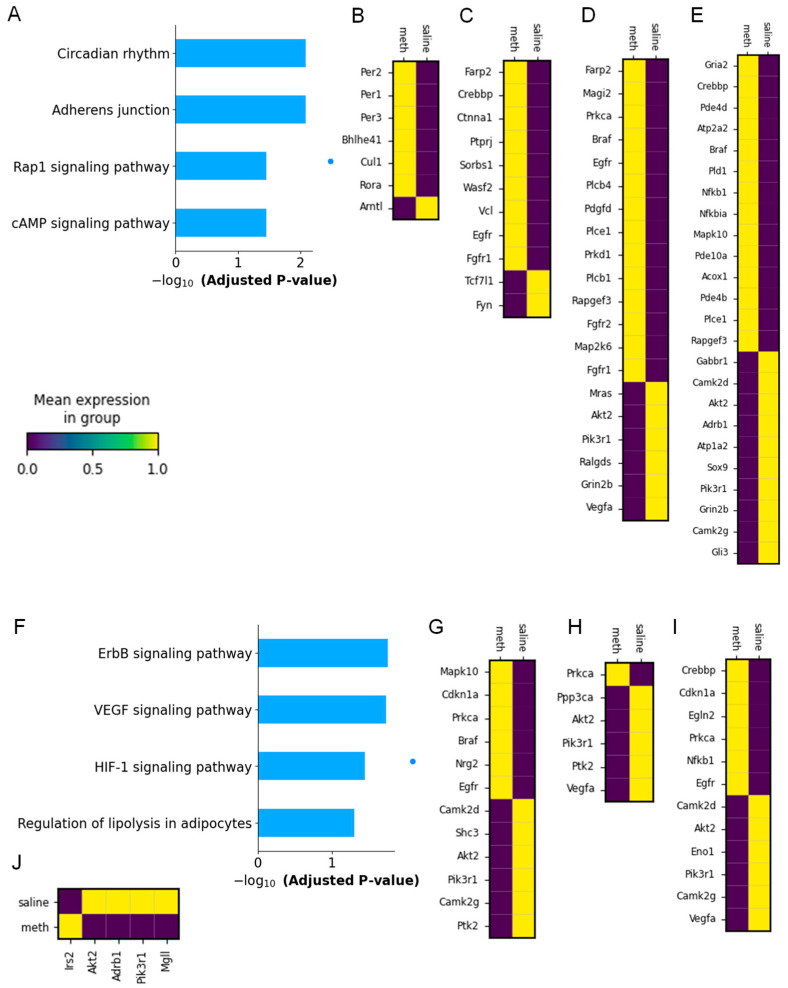
Meth-induced distinct molecular pathways in astrocyte clusters. (**A**) KEGG pathway enrichment analysis of astrocyte clusters in saline- and Meth-administered groups. Heatmap showing DEGs in (**B**) circadian rhythm, (**C**) adherens junction, (**D**) Rap1 signaling pathway, and (**E**) cAMP signaling pathway in saline- and Meth-administered groups. Meth-induced downregulation of distinct molecular pathways in astrocyte clusters. (**F**) KEGG pathway enrichment analysis of astrocyte clusters in saline- and Meth-administered groups. Heatmap showing DEGs in (**G**) ErbB signaling pathway, (**H**) VEGF signaling pathway, (**I**) HIF-1 signaling pathway, and (**J**) regulation of lipolysis in adipocytes in saline- and Meth-administered groups.

We then investigated the detailed molecular mechanism observed in the glial cells isolated from the saline- and Meth-administered wild-type mice. While Kyoto Encyclopedia of Genes and Genomes (KEGG) pathway analysis provided the molecular functions involved in the glial cells after Meth administration, it is crucial to identify the detailed DEGs associated with Meth addiction. In the astrocytes, circadian rhythm, the adherens junction, the Rap1 signaling pathway, and the cAMP signaling pathway were the top four upregulated molecular signaling pathways associated with upregulated DEGs in the astrocytic cell cluster population (Figure 3A). We identified seven DEGs in astrocytes following Meth administration, with six significantly upregulated key circadian-related genes, such as *Per1*, *Per2*, *Per3*, *Bhlhe41*, *Cul1*, and *Rora*, and one downregulated gene, *Amt1* (Figure 3B). Eleven DEGs associated with the adherens junction in the astrocytes that were dysregulated, include nine significantly upregulated genes, such as *Farp2*, *Crebbp*, *Ctnna1*, *Ptprj*, *Sorbs1*, *Wasf2*, *Vcl*, *Egfr*, and *Fgfr1*, and two downregulated genes (*Tcf7l1* and *Fyn*) (Figure 3C). In addition, twenty-one Rap 1 signaling pathway-associated DEGs were significantly dysregulated in the astrocytes in the Meth-administered wild-type mice, with *Farp2*, *Magi2*, *Prkca*, *Braf*, *Egfr*, *Plcb4*, *Pdgfd*, *Plce1*, *Prkd1*, *Plcb1*, *Rapgef3*, *Fgfr2*, *Map2k6*, and *Fgfr1* revealed to be upregulated, and *Mras*, *Akt2*, *Pik3r1*, *Ralgds*, *Grin2b*, and *Vegfa* observed to be downregulated (Figure 3D). Thirteen DEGs (*Gria2*, *Crebbp*, *Atp2a2*, *Braf*, *Pld1*, *Nfkb1*, *Nfkbia*, *Mapk10*, *Pde10a*, *Acox1*, *Pde4b*, *Plce1*, and *Rapgef3*) associated with the cAMP signaling pathway were shown to be upregulated, and ten downregulated DEGs (*Gabbr1*, *Camk2d*, *Atk2*, *Adrb1*, *Atp1a2*, *Sox9*, *Pik3r1*, *Grin2b*, *Camk2g*, and *Gil3*) were found in the astrocyte cluster population of Meth-administered wild-type mice (Figure 3E).

Additionally, KEGG pathway enrichment analysis revealed that the top four downregulated DEGs in the astrocyte cluster population of the Meth-treated mice were mainly enriched in the ErbB signaling pathway, the VEGF signaling pathway, the HIF-1 signaling pathway, and the regulation of lipolysis in adipocytes (Figure 3F). Interestingly, we identified twelve significant DEGs associated with the ErbB signaling pathway in the astrocytes after Meth administration, with six upregulated (*Mapk10*, *Cdkn1a*, *Prkca*, *Braf*, *Nrg2*, and *Egfr*) and six downregulated (*Camk2d*, *Shc3*, *Akt2*, *Pik3r1*, *Camk2g*, and *Ptk2*) genes (Figure 3G). We observed six significant DEGs associated with the VEGF signaling pathway in the astrocytes in the Meth-administered wild-type mice, comparing one upregulated (*Prkca*) and five downregulated genes (*Ppp3ca*, *Akt2*, *Pik3r1*, *Ptk2*, and *Vegfa*) (Figure 3H). The DEGs associated with HIF-1 signaling pathway dysregulation in the astrocyte clusters of the Meth-administered wild-type mice include six upregulated (*Crebbp*, *Cdkn1a*, *Egln2*, *Prkca*, *Nfkb1*, and *Egfr*) and six downregulated (*Camk2d*, *Akt2*, *Eno1*, *Pik3r1*, *Camk2g*, and *Vegfa*) genes (Figure 3I). Lastly, we observed five significant DEGs associated with the regulation of lipolysis in the astrocyte cluster population of the Meth-administered mice, with *insulin receptor substrate 2 (Irs2)* upregulated and *Akt2*, *Adrb1*, *Pik3r1*, and *Mgll* downregulated (Figure 3J).

### 2.3. Dysregulated Molecular Pathways in Microglia After Meth Administration

**Figure 4:**
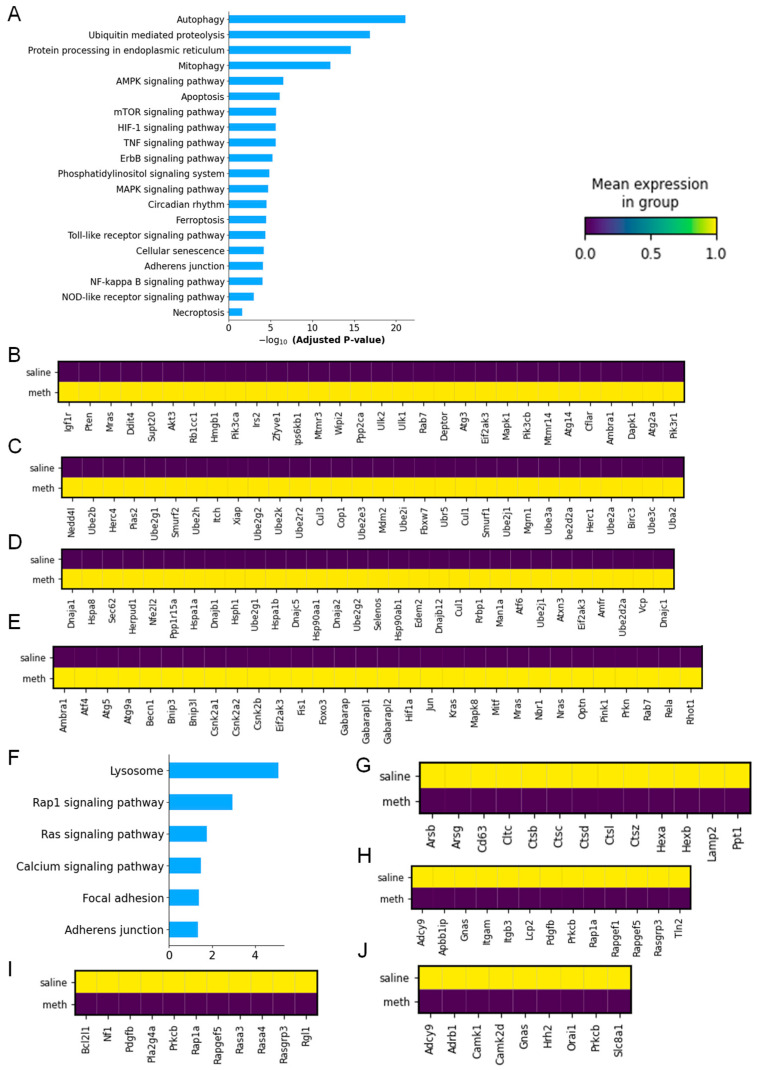
Meth-induced distinct molecular pathways in microglia clusters. (**A**) KEGG pathway enrichment analysis of microglia clusters in saline- and Meth-administered groups. Heatmap showing DEGs in (**B**) autophagy, (**C**) ubiquitin-mediated proteolysis, (**D**) protein processing of endoplasmic reticulum, and (**E**) mitophagy in saline- and Meth-administered groups. Meth-induced downregulation of distinct molecular pathways in microglia clusters. (**F**) KEGG pathway enrichment analysis of microglia clusters in saline- and Meth-administered groups. Heatmap showing DEGs in (**G**) lysosomes, (**H**) Rap1 signaling pathway, (**I**) Ras signaling pathway, and (**J**) calcium signaling pathway in saline- and Meth-administered groups.

To determine the detailed molecular mechanism observed in the microglia clusters isolated from the saline- and Meth-administered wild-type mice, we employed KEGG enrichment pathway analysis. We observed that the top four upregulated molecular pathways induced in the microglia by Meth include autophagy, ubiquitin-mediated proteolysis, the protein processing of endoplasmic reticulum, and mitophagy (Figure 4A).

We identified 86 autophagy-associated DEGs in the microglia clusters after Meth administration, which *include Rab7*, *Mtmr14*, *Pten*, *Irs2*, *Pik3cb*, *Hmgb1*, *Zfyve1*, *Igf1r*, *Stk11*, *Deptor*, *Akt2*, *Rb1cc1*, *Akt3*, *Akt1*, *Prkaca*, *Map3k7*, *Prkacb*, *Snap29*, *Trp53inp2*, *Sh3glb1*, *Atg3*, *Gabarapl2*, *Rubcn*, *Gabarapl1*, *Map2k2*, *Dapk1*, *Pdpk1*, *Atg9a*, *Prkcd*, *Dapk3*, *Atg10*, *Tsc1*, *Wipi2*, *Atg14*, *Atg13*, *Atg12*, *Ern1*, *Vamp8*, *Mras*, *Rragc*, *Pik3ca*, *Traf6*, *Ddit4*, *Akt1s1*, *Atg4c*, *Ulk2*, *Atg4b*, *Ulk1*, *Atg4a*, *Pik3c3*, *Raf1*, *Atg4d*, *Becn1*, *Prkaa1*, *Mtmr3*, *Itpr1*, *Pik3r1*, *Ambra1*, *Hif1a*, *Gabarap*, *Camkk2*, *Ppp2ca*, *Supt20*, *Ppp2cb*, *Nras*, *Mapk8*, *Atg101*, *Mapk1*, *Atg7*, *Atg5*, *Mapk3*, *Igbp1*, *Uvrag*, *Bad*, *Bnip3*, *Eif2ak3*, *Eif2ak4*, *Cflar*, *Mtor*, *Vmp1*, *Rps6kb1*, *Atg1*, *Rheb*, *Atg2a*, *Kras*, and *Atg2b* (Figure 4B and Figure S3A). The key molecular markers associated with microglia cell-mediated ubiquitin-mediated proteolysis (83 DEGs) included *Ube3c*, *Ube2d3*, *Keap1*, *Ube2d1*, *Ube3a*, *Cblb*, *Ube2z*, *Ube2d2a*, *Ube2l3*, *Herc4*, *Herc1*, *Ube2q1*, *Ube2q2*, *Cdc26*, *Cdc27*, *Elob*, *Eloc*, *Btrc*, *Pias4*, *Anapc7*, *Fbxw7*, *Ube2e3*, *Siah1a*, *Ube2e1*, *Ube4a*, *Ube4b*, *Pias2*, *Rbx1*, *Pias1*, *Ddb2*, *Cdc34*, *Ube2r2*, *Traf6*, *Anapc5*, *Birc6*, *Anapc1*, *Birc2*, *Anapc2*, *Birc3*, *Prkn*, *Anapc13*, *Cul5*, *Uba6*, *Mgrn1*, *Cul3*, *Cul2*, *Cul1*, *Nedd4l*, *Xiap*, *Rnf7*, *Ube2j2*, *Rchy1*, *Cbl*, *Anapc10*, *Ube2j1*, *Anapc11*, *Socs3*, *Cop1*, *Ubr5*, *Vhl*, *Ube2h*, *Ube2i*, *Ppil2*, *Ube2b*, *Smurf2*, *Smurf1*, *Huwe1*, *Ube2g1*, *Wwp2*, *Ube2a*, *Ube2g2*, *Cul4a*, *Ube2w*, *Itch*, *Ube2n*, *Mdm2*, *Uba2*, *Uba1*, *Trip12*, *Ube2k*, *Ube2m*, and *Cul4b* (Figure 4C and Figure S3B).

In addition, we identified 89 DEGs associated with the protein processing of endoplasmic reticulum induced in the microglia clusters after Meth administration (Figure 4D and Figure S3C). These DEGs are *Hsp90ab1*, *Tusc3*, *Ube2d3*, *Ngly1*, *Ube2d1*, *Ube2d2a*, *Herpud1*, *Sec61a1*, *Man1a2*, *Sec61g*, *Ufd1*, *Sec61b*, *Sec62*, *Ubxn6*, *Map3k5*, *Sec63*, *Hsp90aa1*, *Ssr4*, *Ssr3*, *Dnajb12*, *Ube4b*, *Rad23b*, *Ddost*, *Rbx1*, *Ern1*, *Dnajc3*, *Man1a*, *Dnajc1*, *Selenos*, *Dnajc5*, *Plaa*, *Preb*, *Man1b1*, *Atf6*, *Atf4*, *Prkn*, *Ppp1r15a*, *Vcp*, *Sec23a*, *Sar1a*, *Atf6b*, *Sar1b*, *Hspa4l*, *Sel1l*, *Cul1*, *Derl2*, *Rrbp1*, *Ube2j2*, *Ube2j1*, *Dnajb2*, *Atxn3*, *Dnajb1*, *Erlec1*, *Mapk8*, *Hsph1*, *Os9*, *Lman2*, *Bag1*, *Ubqln1*, *Map2k7*, *Sec23b*, *Uggt1*, *Edem3*, *Bcap31*, *Hspa8*, *Mbtps1*, *Xbp1*, *Sec24b*, *Sec24a*, *Hspa5*, *Elf2ak1*, *Amfr*, *Edem2*, *Eif2ak3*, *Ube2g1*, *Elf2ak4*, *Ube2g2*, *Dnaja1*, *Nsfl1c*, *Dnajc10*, *Dnaja2*, *Bax*, *Stt3b*, *P4hb*, *Sec24d*, *Rnf185*, *Hspa1b*, *Nfe2l2*, and *Hspa1a*. Lastly, we found that 44 DEGs associated with mitophagy induced in the microglia clusters after Meth administration, which are *Prkn*, *Becn1*, *Rab7*, *Usp15*, *Src*, *Ambra1*, *Foxo3*, *Hif1a*, *Rela*, *Gabarap*, *Nras*, *Mapk8*, *Tbk1*, *Rhot1*, *Ubb*, *Tbc1d17*, *Tbc1d15*, *Atg5*, *Usp8*, *Gabarapl2*, *Bnip3l*, *Fls1*, *Gabarapl1*, *Jun*, *Csnk2a1*, *Bnip3*, *Atg9a*, *Tfe3*, *Csnk2a2*, *Elf2ak3*, *Mitf*, *Pink1*, *Mras*, *Sp1*, *Trp53*, *Csnk2b*, *Nbr1*, *Tax1bp1*, *Tomm7*, *Ulk1*, *Kras*, *Sqstm1*, *Optn*, and *Atf4* (Figure 4E and Figure S3D).

KEGG pathway enrichment analysis revealed that the top four downregulated DEGs in the microglia clusters between the saline and Meth-administered mice were mainly enriched in lysosomes, the Rap1 signaling pathway, the Ras signaling pathway, and the calcium signaling pathway (Figure 4F). Interestingly, 13 DEGs were identified to be induced in the microglia clusters related to lysosome dysregulation in the Meth-administered mice, and the genes significantly downregulated are *Cd63*, *Hexb*, *Ctsz*, *Cltc*, *Hexa*, *Ctsl*, *Lamp2*, *Ppt1*, *Arsg*, *Ctsd*, *Arsb*, *Ctsc*, and *Ctsb* (Figure 4G). We found 13 DEGs that were significantly reduced in the microglia clusters by Meth and associated with the Rap1 signaling pathway, which are *Itgam*, *Prkcb*, *Itgb3*, *Pdgfb*, *Rasgrp3*, *Apbb1ip*, *Adcy9*, *Rap1a*, *Rapgef1*, *Gnas*, *Lcp2*, *Tln2*, and *Rapgef5* (Figure 4H). The number of key molecular genes associated with the Ras signaling pathway was significantly decreased in the microglia clusters by Meth, include *Rap1a*, *Rasa3*, *Prkcb*, *Pdgfb*, *Nf1*, *Pla2g4a*, *Rgl1*, *Rapgef5*, *Rasgrp3*, and *Bcl2l1* (Figure 4I). We noted nine DEGs downregulated in the microglia clusters were associated with the calcium signaling pathway when comparing the saline and Meth-administered mice. The genes downregulated are *Camk2d*, *Adcy9*, *Prkcb*, *Hrh2*, *Gnas*, *Orai1*, *Adrb1*, *Camk1*, and *Slc8a1* (Figure 4J).

### 2.4. Dysregulated Molecular Pathways in Oligodendrocytes After Meth Administration

**Figure 5:**
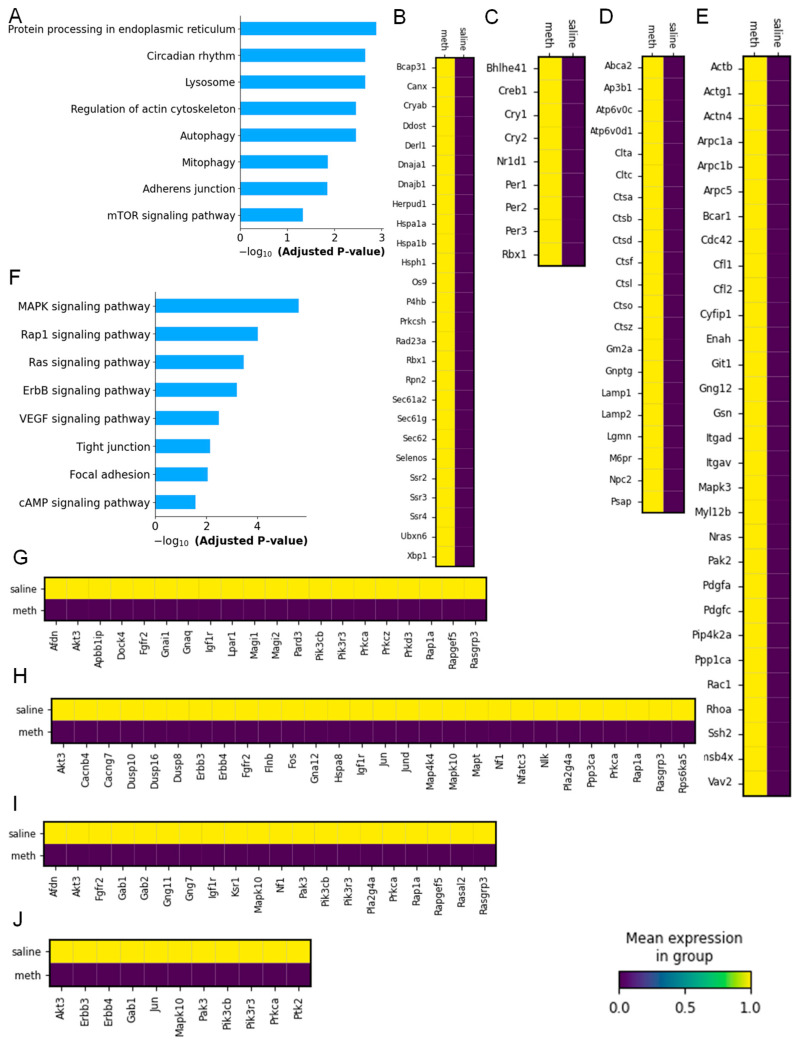
Meth-induced distinct molecular pathways in oligodendrocyte clusters. (**A**) KEGG pathway enrichment analysis of oligodendrocyte clusters in saline- and Meth-administered groups. Heatmap showing DEGs in (**B**) protein processing of endoplasmic reticulum, (**C**) circadian rhythm, (**D**) lysosomes, and (**E**) regulation of actin cytoskeleton in saline- and Meth-administered groups. Meth-induced downregulation of distinct molecular pathways in oligodendrocyte clusters. (**F**) KEGG pathway enrichment analysis of oligodendrocyte clusters in saline- and Meth-administered groups. Heatmap showing DEGs in (**G**) MAPK signaling pathway, (**H**) Rap1 signaling pathway, (**I**) Ras signaling pathway, and (**J**) ErbB signaling pathway in saline- and Meth-administered groups.

We employed KEGG enrichment pathway analysis to explore the detailed molecular mechanisms observed in the oligodendrocyte clusters isolated from the saline- and Meth-administered wild-type mice. We categorized and observed that the top four upregulated molecular pathways induced in the oligodendrocytes by Meth include the protein processing of endoplasmic reticulum, the circadian rhythm, lysosomes, and the regulation of the actin cytoskeleton (Figure 5A). We observed that the 26 DEGs induced in the oligodendrocyte cluster by Meth are *Rpn2*, *Prkcsh*, *Derl1*, *Herpud1*, *Sec61a2*, *Dnajb1*, *Hsph1*, *Os9*, *Sec61g*, *Sec62*, *Ubxn6*, *Bcap31*, *Xbp1*, *Ssr4*, *Ssr2*, *Ssr3*, *Rad23a*, *Ddost*, *Rbx1*, *Dnaja1*, *Selenos*, *Canx*, *P4hb*, *Cryab*, *Hspa1b*, and *Hspa1a* (Figure 5B). Just like the astrocytes, we found nine DEGs induced in the oligodendrocytes that are associated with the circadian rhythm in Meth, including *Per2*, *Per1*, *Per3*, *Creb1*, *Cry2*, *Bhlhe41*, *Cry1*, *Nr1d1*, and *Rbx1* (Figure 5C). While there are fewer DEGs (21 genes) observed to be associated with lysosomes in the oligodendrocyte cluster induced by Meth when compared to those in the microglia cluster, the genes upregulated are *Ctsa*, *Abca2*, *Ctsz*, *Cltc*, *M6pr*, *Clta*, *Ap3b1*, *Gnptg*, *Ctso*, *Lamp1*, *Gm2a*, *Ctsl*, *Npc2*, *Lamp2*, *Psap*, *Ctsf*, *Atp6v0d1*, *Ctsd*, *Atp6v0c*, *Lgmn*, and *Ctsb* (Figure 5D). Oligodendrocytes demonstrate significant involvement in the regulation of the actin cytoskeleton as the 30 DEGs that were increased are *Cyflp1*, *Arpc1b*, *Arpc1a*, *Pdgfa*, *Actb*, *Myl12b*, *Actg1*, *Cdc42*, *Nras*, *Tmsb4x*, *Cfl2*, *Pdgfc*, *Cfl1*, *Plp4k2a*, *Itgav*, *Rac1*, *Pak2*, *Git1*, *Mapk3*, *Gsn*, *Actn4*, *Arpc5*, *Gng12*, *Ssh2*, *Rhoa*, *Vav2*, *Ppp1ca*, *Enah*, *Itgad*, and *Bcar1* (Figure 5E).

Utilizing KEGG pathway enrichment analysis, we identified that the top four downregulated DEGs in the oligodendrocyte cluster between the saline and Meth-administered mice were mainly enriched in the MAPK signaling pathway, the Rap1 signaling pathway, the Ras signaling pathway, and the ErbB signaling pathway (Figure 5F). We found that Meth significantly downregulated 28 DEGs associated with the MAPK signaling pathways, and they are *Nlk*, *Dusp16*, *Igf1r*, *Rasgrp3*, *Ppp3ca*, *Cacng7*, *Rps6ka5*, *Rap1a*, *Dusp10*, *Erbb3*, *Erbb4*, *Akt3*, *Gna12*, *Flnb*, *Map4k4*, *Hspa8*, *Jun*, *Jund*, *Nfatc3*, *Pla2g4a*, *Prkca*, *Fos*, *Dusp8*, *Mapk10*, *Cacnb4*, *Nf1*, *Mapt*, and *Fgfr2* (Figure 5G). In contrast to the astrocyte clusters, we identified 20 DEGs, which include *Magl1*, *Dock4*, *Magl2*, *Lpar1*, *Pik3r3*, *Prkca*, *Pik3cb*, *Prkcz*, *Gnal1*, *Igf1r*, *Rasgrp3*, *Apbb1Ip*, *Afdn*, *Rap1a*, *Prkd3*, *Pard3*, *Gnaq*, *Akt3*, *Rapgef5*, and *Fgfr2*, associated with the Rap1 signaling pathway to be significantly decreased in the oligodendrocytes by Meth (Figure 5H). Oligodendrocytes showed significant involvement in the Ras signaling pathway, as Meth downregulated 20 DEGs, and these include *Ksr1*, *Gab1*, *Pik3r3*, *Pla2g4a*, *Prkca*, *Gab2*, *Rasal2*, *Pik3cb*, *Gng11*, *Igf1r*, *Rasgrp3*, *Mapk10*, *Afdn*, *Rap1a*, *Gng7*, *Akt3*, *Nf1*, *Rapgef5*, *Pak3*, and *Fgfr2* (Figure 5I). Interestingly, the oligodendrocytes showed a similar pattern as the astrocytes, as the 11 DEGs downregulated in the ErbB signaling pathway by Meth are *Mapk10*, *Jun*, *Erbb3*, *Erbb4*, *Akt3*, *Gab1*, *Pik3r3*, *Prkca*, *Pik3cb*, *Pak3*, and *Ptk2* (Figure 5J).

### 2.5. Identification of Transcription Factors in Glial Cells After Meth Administration

**Figure 6:**
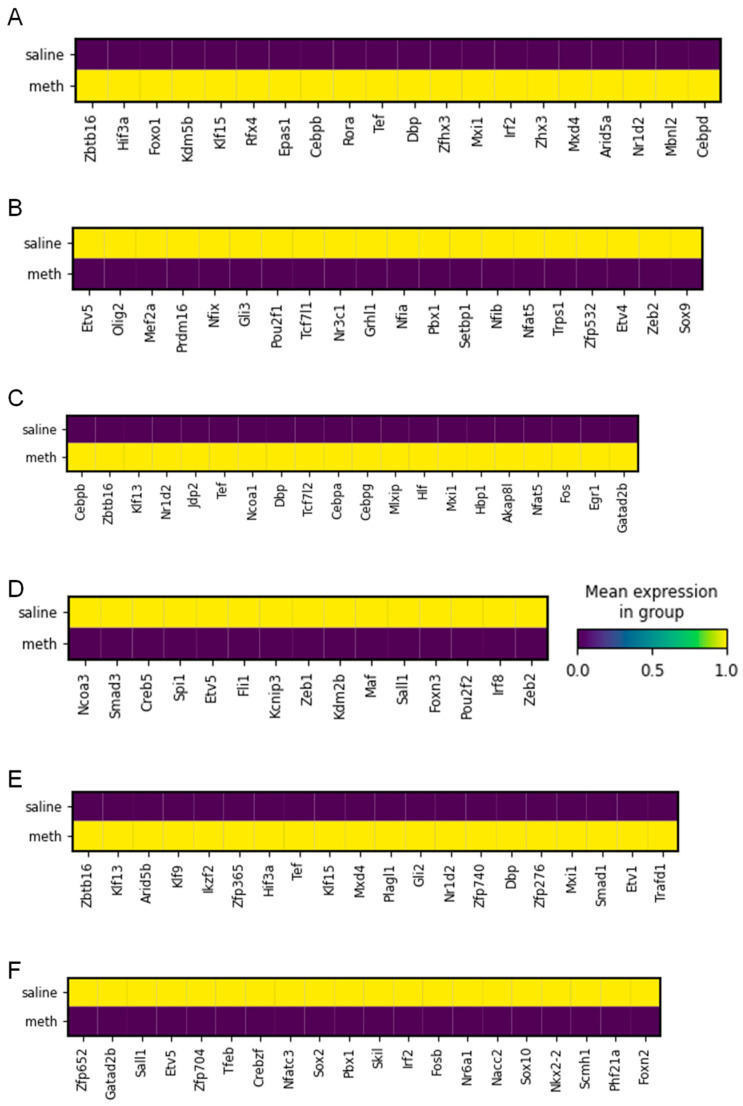
Transcription factor changes associated with Meth exposure. Heatmap of top 25 (**A**) upregulated and (**B**) downregulated transcription factor DEGs in astrocyte clusters in saline- and Meth-administered groups. Heatmap of top 25 (**C**) upregulated and (**D**) downregulated transcription factor DEGs in microglia clusters in saline- and Meth-administered groups. Heatmap of top 25 (**E**) upregulated and (**F**) downregulated transcription factor DEGs in oligodendrocyte clusters in saline- and Meth-administered groups.

Next, we wanted to determine the various transcription factors (TFs) that control the transcription rate of the DEGs observed in the glial cell clusters after Meth administration. This investigation revealed large variability in the molecular phenotypes, highlighting variations in transcription factors in the glial cell clusters after Meth administration. Here, we implemented TF identification using the single-cell libraries and analyzed the astrocyte-, microglia-, and oligodendrocyte-captured cells that passed quality control. We identified the top twenty upregulated differentially expressed TFs in the astrocyte clusters in the Meth-administered mice as *Zbtb16*, *Hif3a*, *Foxo1*, *Kdm5b*, *KIf15*, *Rfx4*, *Epas1*, *Cebpp*, *Rora*, *Tef*, *Dbp*, *Zfhx3*, *Mxi1*, *Irf2*, *Zhx3*, *Mxd4*, *Arid5a*, *Nr1d2*, *Mbnl2*, and *Cebpd* according to their adjusted *p*-value (Figure 6A). In addition, we characterized the top twenty downregulated differentially expressed TFs in the astrocyte clusters in the Meth-administered mice as *Etv5*, *Olig2*, *Mef2a*, *Prdm16*, *Nfix*, *Gli3*, *Pou2f1*, *Tcf7l1*, *Nr3c1*, *Grhl1*, *Nfia*, *Pbx1*, *Setbp1*, *Nfib*, *Nfat5*, *Trps1*, *Zfp532*, *Etv4*, *Zeb2*, and *Sox9* (Figure 6B).

Notably, we observed the top twenty upregulated differentially expressed TFs induced in the microglia clusters by Meth are *Cebpb*, *Zbtb16*, *Kif13*, *Nr1d2*, *Jdp2*, *Tef*, *Ncoa1*, *Dbp*, *Tcf7l2*, *Cebpa*, *Cebpg*, *Mlxip*, *Hif*, *Mxi1*, *Hbp1*, *Akap8l*, *Nfat5*, *Fos*, *Egr1*, and *Gatad2b* (Figure 6C). In addition, we found the repression of differentially expressed TFs, such as *Ncoa3*, *Smad3*, *Creb5*, *Spi1*, *Etv5*, *Fli1*, *Kcnip3*, *Zeb1*, *Kdm2b*, *Maf*, *Sall1*, *Foxn3*, *Pou2f2*, *Irf8*, and *Zeb2*, in the microglia by Meth (Figure 6D). Interestingly, we observed *Zbtb16*, *Klf13*, *Arid5b*, *Klf9*, *Ikzf2*, *Zfp365*, *Hif3a*, *Tef*, *Klf15*, *Mxd4*, *Plagl1*, *Gli2*, *Nr1d2*, *Zfp740*, *Dbp*, *Zfp276*, *Mxi1*, *Smad1*, *Etv1*, and *Trafd1* were the top twenty differentially expressed genes upregulated in the oligodendrocytes by Meth (Figure 6E). As well, we highlight *Zfp652*, *Gatad2b*, *Sall1*, *Etv5*, *Zfp704*, *Tfeb*, *Crebzf*, *Nfatc3*, *Sox2*, *Pbx1*, *Skil*, *Irf2*, *Nr6a1*, *Nacc2*, *Sox10*, *Nkx2-2*, *Scmh1*, *Phf21a*, and *Foxn2* as the top twenty downregulated DEGs in the oligodendrocytes by Meth (Figure 6F).

### 2.6. Functional Characterization of Glial Cell Clusters After Meth Administration

**Figure 7:**
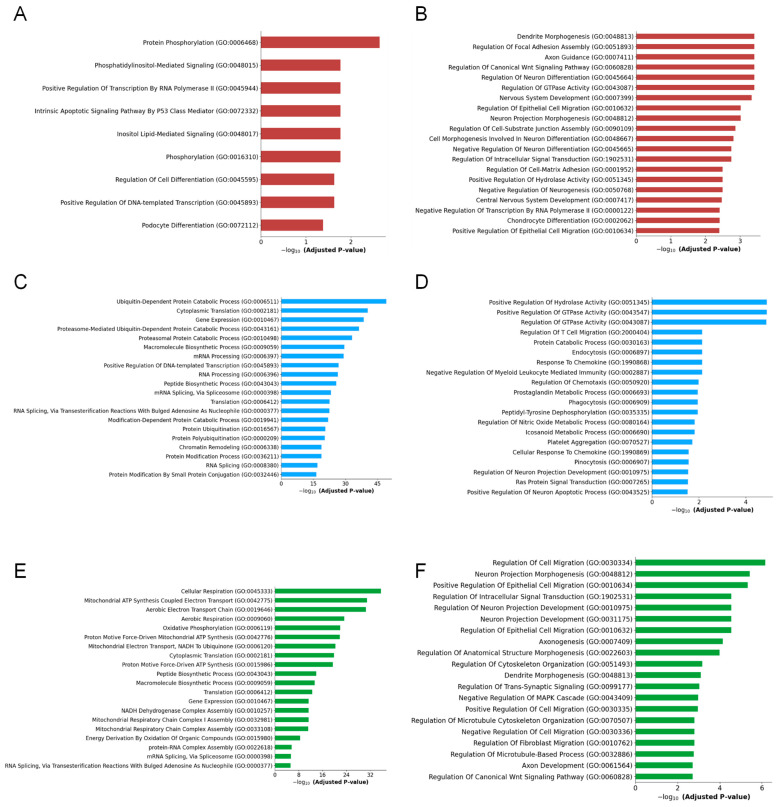
Meth-induced distinct biological changes in specific glial cell clusters. (**A**) Upregulated and (**B**) downregulated GO biological component enrichment analysis of astrocyte clusters in saline- and Meth-administered groups. (**C**) Upregulated and (**D**) downregulated GO biological component enrichment analysis of microglia clusters in saline- and Meth-administered groups. (**E**) Upregulated and (**F**) downregulated GO biological component enrichment analysis of oligodendrocyte clusters in saline- and Meth-administered groups.

To determine the functional roles of the DEGs in the glial cell clusters in the Meth-administered wild-type mice, we performed Gene Ontology (GO) annotation using DAVID version 2021 and obtained enriched terms for the biological process. The GO biological process classification system revealed that the DEGs in the astrocytes could be enriched according to their functional properties. The top four biological processes uncovered revealed the enrichment of the upregulated DEGs in the astrocytes after Meth administration are related to protein phosphorylation, phosphatidylinositol-mediated signaling, the positive regulation of transcription by RNA polymerase ll, and the intrinsic apoptotic signaling pathway by the P53 class mediator (Figure 7A). Conversely, the top four biological processes for the downregulated DEGs in the astrocytes after Meth administration revealed the enrichment of processes related to dendrite morphogenesis, the regulation of the focal adhesion assembly, axon guidance, and the regulation of the canonical Wnt signaling pathway (Figure 7B).

Our analysis of the microglia clusters indicated that the top four enriched biological process, based on upregulated DEGs, are ubiquitin-dependent protein catabolic process, cytoplasmic translation, gene expression, and proteasome-mediated ubiquitin-dependent protein catabolic process (Figure 7C). Additionally, the analysis of the top four biological processes revealed that the downregulated DEGs were enriched in processes such as the positive regulation of hydrolase activity, the positive regulation of GTPase activity, the regulation of GTPase activity, and the regulation of T cell migration (Figure 7D). Surprisingly, biological process analysis revealed the enrichment of the upregulated DEGs in the oligodendrocytes after Meth administration are related to cellular respiration, mitochondrial ATP synthesis-coupled electron transport, the aerobic electron transport chain, and aerobic respiration (Figure 7E). However, biological process analysis revealed the enrichment of the downregulated DEGs in the oligodendrocytes induced by Meth are the regulation of cell migration, neuron projection morphogenesis, the positive regulation of epithelial cell migration, and the regulation of intracellular signal transduction (Figure 7F).

## 3. Discussion

Methamphetamine is a potent neurotoxic stimulant with a strong potential for addiction, affecting over 36 million individuals globally as of 2021 [23]. While methamphetamine addiction is a significant global issue, the precise molecular mechanisms responsible for the structural and biochemical changes it induces in the brain remain unclear. Methamphetamine alters multiple biological processes, including oxidative stress, synaptic plasticity, the cell death pathways (e.g., apoptosis and necroptosis), neuroinflammation, and the dysregulation of neurotransmitter systems, such as dopamine and serotonin, as well as immune responses via cytokine modulation [24,25,26,27,28]. Glial cells, particularly microglia and astrocytes, play a critical role in modulating neuronal activity and contributing to methamphetamine-induced neurotoxicity [28]. However, the specific roles and transcriptomic changes in glial cells, like microglia, astrocytes, and oligodendrocytes, in the context of methamphetamine exposure remain unexplored [18].

In this study, we employed scRNA-seq to investigate gene expression changes in the cortical region of the brains of methamphetamine-administered mice. We aimed to elucidate the involvement of various signaling pathways across heterogeneous cell populations. Our results identified distinct glial clusters, with microglia, astrocytes, and oligodendrocytes showing the highest number of DEGs. This finding aligns with previous research highlighting the significant involvement of non-neuronal cells, particularly glia, in substance use disorders. For instance, previous studies have demonstrated more DEGs in glial cells compared to those in neurons in alcohol dependence [29]. Similarly, transcriptional responses have been observed in astrocytes and oligodendrocytes in the nucleus accumbens of alcohol and morphine-administered mice, respectively [20,22]. We also observed a reduction in cell numbers across different cortical cell types in the methamphetamine-administered mice compared with those of the controls.

Astrocytes are the key constituents in the formation and maintenance of the blood–brain barrier, providing a highly controlled framework for the CNS microenvironment [30,31]. Additionally, astrocytes secrete neurotrophic factors that regulate synaptogenesis, neuronal differentiation, and survival. They also modulate synaptic transmission through neurotransmitter release and clearance [32,33,34,35,36,37,38]. While these functions are essential for CNS homeostasis, the dysregulation of astrocytic activities at the molecular and functional levels has been implicated in several CNS disorders [39,40,41].

In our study, Meth administration significantly impaired the astrocytic pathways, including the circadian rhythm, the adherens junction, Rap1 signaling, cAMP signaling, ErbB signaling, VEGF signaling, HIF-1 signaling, and the regulation of lipolysis in adipocytes. The physiological relevance of alterations of these pathways in the astrocytes by Meth are relevant in the progression of neuroinflammation, impaired neurovascular support, and structural changes, leading to neuronal damage. These transcriptional alterations in astrocytes may contribute to the progression of Meth addiction. Specifically, the genes associated with circadian rhythms, such as *Per1*, *Per2*, *Per3*, and *Cul1*, were significantly upregulated in the astrocytes isolated from the cortical regions of the Meth-administered mice. The circadian rhythm is known to regulate numerous genes involved in processes like inflammation, redox homeostasis, and the cell cycle, implicating it in diseases ranging from cancer to neurodegeneration [42,43]. The Meth-induced disruption of circadian rhythms may exacerbate addiction and relapse behaviors, as studies have documented links between sleep disturbances and substance abuse, particularly insomnia, in recovering people with an addiction [44,45,46,47].

Interestingly, the adherens junction pathway, which is critical for cell–cell adhesion, actin cytoskeleton regulation, and transcriptional control, was also impacted by Meth exposure [48]. Our study identified the significant downregulation of Tcf7l1, a known nuclear repressor of the Wnt signaling pathway, which regulates cell fate, migration, and polarity [49]. Conversely, we observed the upregulation of *Frap2 (Mtor)* and *Crebbp*, both of which play essential roles in cell proliferation, differentiation, survival, glucose homeostasis, and synaptic plasticity [50,51,52,53,54]. This aligns with the previous reports of Meth-induced CREB phosphorylation in rat striata [55,56], although contrasting studies suggest mTOR inhibition in neuronal cells under certain conditions [57,58,59].

Additionally, the genes involved in the Rap1 and cAMP signaling pathways were significantly altered in the astrocyte clusters. While the role of Rap1 signaling in Meth toxicity is not well documented, its association with guanine nucleotide exchange factors such as Epac and its influence on ERK-mediated transcription are well established in Meth toxicity studies [60,61,62]. Our findings corroborate the data suggesting that CREB activation is a master regulator of EAAT-2 transcription downstream of Meth-induced TAAR1 activation [63]. Meth also significantly downregulated the ErbB signaling pathway, known for its neuroprotective and repair mechanisms [64,65]. This downregulation exacerbates Meth-induced neuronal damage and neuroinflammation by impairing the survival pathways. Similarly, the disruption of VEGF signaling likely occurs through the impact of Meth on upstream ErbB components, further contributing to vascular and neuronal dysfunction [66,67]. The suppression of the HIF-1 signaling pathway, which regulates the cellular hypoxic response, further highlights the neurotoxic potential of Meth. Meth destabilizes HIF-1α, impairing the transcription of protective genes like VEGF and amplifying neurodegeneration and vascular damage [68]. The disruption of lipolysis may contribute to the neuroinflammatory environment and energy deficits observed in Meth-induced neuropathology [69].

In microglia, Meth exposure significantly altered the gene expression related to autophagy, ubiquitin-mediated proteolysis, and lysosomal function. Genes such as *Keap1* and *Cul4b* were notably upregulated, affecting autophagic regulation and proteostasis [70,71]. The dysregulation of *Rap1*, *Ras*, and the calcium signaling pathways further impaired microglial functionality, exacerbating oxidative stress, cytokine production, and synaptic dysfunction [72,73,74]. Interestingly, the existing data have supported evidence that Meth significantly decreased the lysosome–associated proteins such as Lamp2 to aggravate neurotoxicity [73]. The Meth-induced downregulation of genes, such as *Rap1a*, *Rapgef1*, and *Tln2*, affects microglial ability to maintain synaptic interactions and respond effectively to neural injury [74,75]. The altered Rap1 signaling also contributes to impaired microglial chemotaxis, potentially exacerbating neuroinflammation [74,75]. Meth administration also decreases key Ras-associated genes, such as *Rap1a*, *Nf1*, and *Pla2g4a*, thereby impairing microglial proliferation and increasing susceptibility to neurodegeneration [72,76]. The deregulation of Ras signaling in Meth-exposed microglia further amplifies oxidative stress and cytokine production, intensifying neural damage [72,76]. Additionally, the Meth-induced downregulation of *Camk2d*, *Adcy9*, and *Slc8a1* disrupts calcium homeostasis, impairing microglial responsiveness to injury and synaptic modulation. Dysregulated calcium signaling also increases microglial activation and inflammatory cytokine release, contributing to the neurotoxic environment, ultimately impairing neuronal function [77,78].

In the oligodendrocytes, Meth exposure induced the genes associated with ER stress, circadian rhythm, and lysosomal function. This included the upregulation of *Hspa1a* and *Hspa1b*, indicative of unfolded protein response activation, and alterations in circadian genes, like *sPer1*, *Per2*, *Cry1*, and *Cry2*, which disrupt myelin synthesis and energy metabolism [79,80]. Additionally, increased lysosomal activity and disrupted actin cytoskeleton dynamics further compromised oligodendrocyte functionality [81,82]. Our data provided evidence that the Meth-induced disruption of circadian genes, such as *Per1*, *Per2*, *Per3*, *Nr1d1*, *Cry1*, and *Cry2*, alters myelin synthesis and energy metabolism in oligodendrocytes [45,79]. This dysregulation might contribute to the cognitive and behavioral impairments observed in Meth users [83]. Our data reveal that Meth increases lysosomal activity in oligodendrocytes, as evidenced by the upregulation of genes like *Lamp1* and *Cathepsin D*. While enhanced lysosomal activity may initially protect against Meth-induced damage, prolonged stimulation can lead to lysosomal dysfunction and cell death, further degrading myelin integrity [81,82,84]. The Meth-induced upregulation of genes like *RhoA*, *Cfl1*, and *Cfl2* alters cytoskeletal dynamics, impairing oligodendrocyte process extension and myelin sheath formation [85,86,87]. Disrupted actin regulation contributes to compromised oligodendrocyte function and myelination deficits [85,86,87]. In addition, our data observed the downregulation of the Rap1 and Ras signaling pathways in oligodendrocytes by Meth to impair function and proliferation via oxidative stress and cytokine production, thereby exacerbating neuroinflammation and neurotoxicity [72,76]. Also, the ErbB signaling pathway was significantly downregulated in oligodendrocytes by Meth, impairing neuroprotective and repair mechanisms [64,65].

Meth administration has been shown to significantly alter the transcriptional landscape in astrocytes, microglia, and oligodendrocytes with the notable upregulation of *Zbtb16*, *Hif3a*, *Foxo1*, and *Klf9*. These transcription factors are critically involved in diverse cellular processes, suggesting their potential roles in the astrocyte-, microglia- and oligodendrocyte-related responses to Meth-induced neurotoxicity. *Zbtb16 (PLZF)*, a member of the BTB-ZF transcription factor family, is associated with stress response and differentiation and may contribute to the activation of inflammatory and stress-resilient glial phenotypes [88,89,90]. *Hif3a*, a lesser-studied member of the hypoxia-inducible factor family, can modulate HIF-1α activity, indicating a potential role in altering the hypoxic response and energy metabolism under Meth-induced oxidative stress [91,92]. *Foxo1*, a forkhead transcription factor, is central to oxidative stress response and apoptosis; an increase in quantity might reflect the astrocytes’ attempt to adapt to Meth’s pro-oxidative environment, potentially leading to metabolic reprogramming and survival signaling [93,94]. Circadian clock-output transcription factor genes, such as *Dbp* and *Tef*, induced in glial cells by Meth mediate circadian variation in physiology, metabolism, oxidative signaling, and other biological processes [95,96,97]. *Etv5*, a member of the ETS transcription factor family, plays a role in cellular differentiation and the response to growth factors, particularly in the central nervous system; its downregulation may impair glial cell plasticity and neurotrophic support [98,99]. Collectively, the dysregulation of these transcription factors highlights a complex interplay of stress response, metabolic reprogramming, and epigenetic regulation in glial cells, underscoring their pivotal role in the neuropathological effects of Meth.

While the bioinformatic tools were essential for investigating the DEG dataset, it is important to recognize that transcriptomic changes do not always correlate with protein expression or functional outcomes. Relying on transcriptomic data alone could miss post-transcriptional modifications and changes in protein levels, which could be crucial for understanding methamphetamine-induced neurotoxicity. In our study, although a large number of mRNA variants were identified in the glial cells following methamphetamine exposure, further research is nevertheless warranted to explore how these translate into protein variants, signaling changes, and cellular localization. Validation using complementary approaches, such as proteomics, Western blotting, and immunohistochemistry, is critical for confirming the involvement of these genes. Future studies should also explore the effects of methamphetamine over time, using multi-timepoint analysis to capture dynamic gene expression changes during the acute, chronic, and withdrawal phases.

A key limitation of this study is the exclusion of female mice, which restricts the ability to assess potential sex-specific transcriptional responses to Meth-induced neurotoxicity. Female mice were not included to minimize the variability associated with hormonal fluctuations, as these can influence transcriptional outcomes in neuroinflammatory models. While this approach was intended to maintain experimental consistency and address the primary objectives within the resource constraints, it limits the generalizability of our findings to both sexes. Future studies will address this limitation by incorporating both male and female mice to comprehensively evaluate the sex-specific transcriptional changes and their implications in Meth-induced neurotoxicity. This will provide a broader understanding of the molecular mechanisms underlying the effects of Meth on the brain. Also, it is crucial to ensure sufficient animal numbers in each group to obtain statistically reliable results. Investigating additional brain regions, such as the hippocampus and the nucleus accumbens, is also important to comprehensively understand the effects of methamphetamine across the brain. While this study specifically focused glial cells, astrocytes, microglia, and oligodendrocytes, how Meth impairs endothelial cell integrity warrants further studies. Finally, while scRNA-seq was valuable in revealing cell heterogeneity, using single-nuclei RNA sequencing could help us to study the difficult-to-isolate glial cells and neuronal cells. Combining transcriptomics with other omics approaches, such as proteomics or epigenomics, would provide a more integrated view of the molecular mechanisms underlying methamphetamine-induced pathogenesis.

## 4. Materials and Methods

### 4.1. Animal Model and Tissue Collection

Sample size: A total of six male C57BL/6 mice (*n* = 3 per group) were used. Sample size was determined based on the exploratory nature of this study and resource constraints. While this sample size is consistent with preliminary scRNA-seq studies, the findings should be interpreted as foundational, with plans for larger follow-up studies.

Inclusion and exclusion criteria: Healthy, 10-week-old male C57BL/6 mice were included in this study. Animals showing signs of illness, injury, or weight loss beyond 15% of the baseline were excluded. One Meth-treated sample was excluded during scRNA-seq analysis due to its high mitochondrial RNA content (>10%), indicative of cell stress or death.

Randomization: The mice were randomly assigned to either the Meth-treated or saline-treated groups using an alternating assignment protocol. This ensured unbiased group allocation.

Blinding: Blinding was implemented during data analysis to reduce bias. The researchers the analyzing scRNA-seq data were blinded to the treatment groups.

Experimental animals: Six healthy male C57BL/6 mice (10 weeks old) were used in this study, housed in accordance with the University of Nebraska Medical Center Institutional Animal Care and Use Committee protocol (Approval Number: #18-030-04-FC). All experimental procedures complied with the Ethics Committee of the University of Nebraska Medical Center. Following euthanasia, the brains were dissected, and the cortical regions were isolated for scRNA-seq. The age group utilized represents young adult humans, which has been widely used in preclinical addiction studies to model the neurotoxic and behavioral effects of psychostimulants [100,101,102].

### 4.2. Methamphetamine Administration

The wild-type mice were acclimated to methamphetamine through an escalating dosage protocol [103,104]. Methamphetamine (2–10 mg/kg in sterile saline) was administered intraperitoneally over five days, starting at 2 mg/kg and increasing to 10 mg/kg. After the acclimation period, the mice were maintained on 10 mg/kg methamphetamine for 28 days. The control mice received daily saline injections. After treatment, all the mice were anesthetized with isoflurane and euthanized via cervical dislocation. Six male mice were utilized for this experiment.

### 4.3. 10x Chromium scRNA-Seq

scRNA-seq was performed on cortical brain tissues using the Adult Brain Dissociation Kit (Cat No. 130-107-677, Miltenyi Biotec Inc. Auburn, CA, USA) per the manufacturer’s instructions. Briefly, the brain cerebral cortex tissues were homogenized, resuspended in enzyme mixes, and dissociated using a gentleMACS Octo Dissociator. Cells were filtered and processed for debris and red blood cell removal. Cell viability and number were assessed using trypan blue staining. We generated scRNA-seq libraries using the 10x Genomics Chromium Single Cell 3’ Library and Gel Bead Kit. Approximately 8000 cells were loaded onto the 10x Genomics Chromium GEM chip for cell capture and library preparation. Libraries were sequenced using Illumina NextSeq550 and NovaSeq6000. The data have been deposited in the NCBI with the SRA reference number PRJNA1180116.

### 4.4. Bioinformatic Analysis

The raw sequencing data from the Illumina NextSeq550 and NovaSeq6000 platforms (5200 Illumina Way, San Diego, CA, USA) were first demultiplexed, and then converted to FASTQ files using CellRanger 2.0.0 (10x Genomics). The sequences were aligned to the mouse reference genome (GRCm39-2024-A) using the CellRanger count pipeline (CellRanger-8.0.0), which generated a gene-barcode matrix for each sample. The resulting raw gene expression matrices were then processed using several quality control (QC) and normalization steps to ensure high data quality and accurate downstream analysis.

To remove ambient RNA contamination and other technical artifacts, the cellbender 0.3.0 tool was used to subtract noise from the raw gene expression data [105]. After removal, quality control and further downstream processing was performed using the Scanpy 1.10.1 Python package [106]. Cells were filtered based on the total UMI counts, gene counts, and mitochondrial transcript fraction. Cells with extreme log-transformed total counts or gene counts (greater or less than five median absolute deviations from the median) were excluded from further processing. Additionally, cells with more than 10% of their RNA reads derived from mitochondrial transcripts were removed, as this indicates potential cell stress or death. This resulted in the removal of one of the Meth-administered mice sample as it failed quality control check and was not utilized for downstream analysis.

To identify and eliminate doublets, we used the scrublet algorithm [107], which assigns doublet scores to each barcode and flags candidate doublets for removal. After these filtering steps, the remaining high-quality cells were retained for downstream analysis.

Normalized gene expression values were obtained using log normalization, where the normalized total count from each cell equal to the median raw UMI count was scaled by the median of total counts in each cell and multiplied by a scaling factor of 10,000 before log transformation. The resulting normalized dataset was then used to compare the methamphetamine and saline-treated groups. The data from both the groups were concatenated using the Scanpy concat function.

Dimensionality reduction was performed using Principal Component Analysis (PCA), followed by Uniform Manifold Approximation and Projection (UMAP) to visualize the high-dimensional scRNA-seq data in two dimensions [108]. Before UMAP, highly-variable genes (HVGs) were identified based on their mean expression and dispersion, and the top 4000 HVGs were selected for analysis. UMAP was then applied to the first 50 principal components from PCA, providing a low-dimensional visualization of the cell populations.

Each cluster was assigned a cell type based on canonical marker genes using the MapMyCells hierarchical annotation tool (Allen Institute for Brain Science and BRAIN Initiative Cell Atlas Network) [109]. Known markers, such as *Tmem119* and *Csf1r* (microglia), *Gfap* and *Slc1a3* (astrocytes), and *Olig1* and *Mog* (oligodendrocytes), were used to annotate the clusters, along with endothelial and other CNS cell types.

Differential gene expression analysis between the methamphetamine- and saline-treated groups was conducted using the Wilcoxon rank-sum test implemented with the Scanpy rank_gene_groups function. The differentially expressed genes (DEGs) were identified based on a Benjamini–Hochberg-adjusted *p*-value < 0.05 to account for multiple comparisons. The DEGs were further stratified by cell type to highlight methamphetamine-induced transcriptional changes specific to the microglia, the astrocytes, and the oligodendrocytes.

GO enrichment analysis and KEGG pathway analysis were performed using the gseapy Python package. Over-representation analysis (ORA) was applied to the DEGs to identify the significantly enriched biological processes, molecular functions, and cellular components. Enrichr was used to explore transcription factor–target interactions and drug-induced gene sets, providing additional insights into the regulatory mechanisms underlying methamphetamine-mediated changes in gene expression.

### 4.5. Statistical Analysis

An alpha level of 0.05 was used as the threshold for statistical significance in all comparisons between the saline and methamphetamine-administered mice across the different cell types. The DEGs were identified using a non-parametric Wilcoxon rank-sum test, which is suitable for comparing gene expression distributions between groups without assuming normality. To control for multiple comparisons, *p*-values were adjusted using the Benjamini–Hochberg correction method, ensuring the robust identification of significant DEGs. For the ORA of GO terms and KEGG pathways, enrichment *p*-values were calculated using a hypergeometric distribution. *p*-values from ORA were further adjusted using the Benjamini–Hochberg method to control the false discovery rate (FDR). Significant enrichment was considered at an adjusted *p*-value (FDR) < 0.05 when comparing the saline and methamphetamine-administered mice across the various cell types.

## 5. Conclusions

Our scRNA-seq data provide comprehensive insights into cell type-specific transcriptional changes in the cortical brains of methamphetamine-administered mice, highlighting the key pathways linked to methamphetamine-related disorders. By analyzing the DEGs across various glial cell populations, we emphasize the role of glial cells, such as microglia, astrocytes, and oligodendrocytes, in mediating neuronal damage in methamphetamine toxicity. Given the challenges in validating therapeutic targets across isolated physiological systems, identifying shared glial cell genes offers promising therapeutic strategies. Our bioinformatics analysis contributes to filling the gaps in understanding the molecular triggers induced by methamphetamine, potentially paving the way for novel interventions targeting methamphetamine-induced immune dysfunction.

## Data Availability

These data will be made available on request.

## Supplementary Materials

The following supporting information can be downloaded at: https://www.mdpi.com/article/10.3390/ijms26020649/s1.
