# Supplementary material for: Single-Cell RNA-Seq Uncovers Robust Glial Cell Transcriptional Changes in Methamphetamine-Administered Mice
Source: Int J Mol Sci. 2025 Jan 14;26(2):649. doi: 10.3390/ijms26020649 (PMC11766323; doi:10.3390/ijms26020649)
Supplement: Supplementary file 1 [file ijms-26-00649-s001.zip › ijms-3401470-supplementary.pdf]

Figure S1.

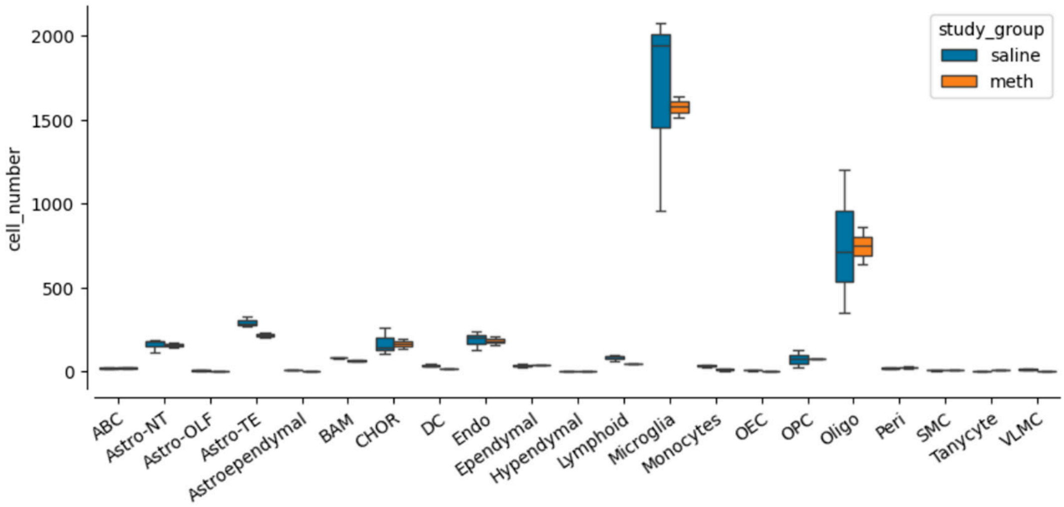

Cell counts of distinct cell types in saline- and methamphetamine-administered mice.

Figure S2.

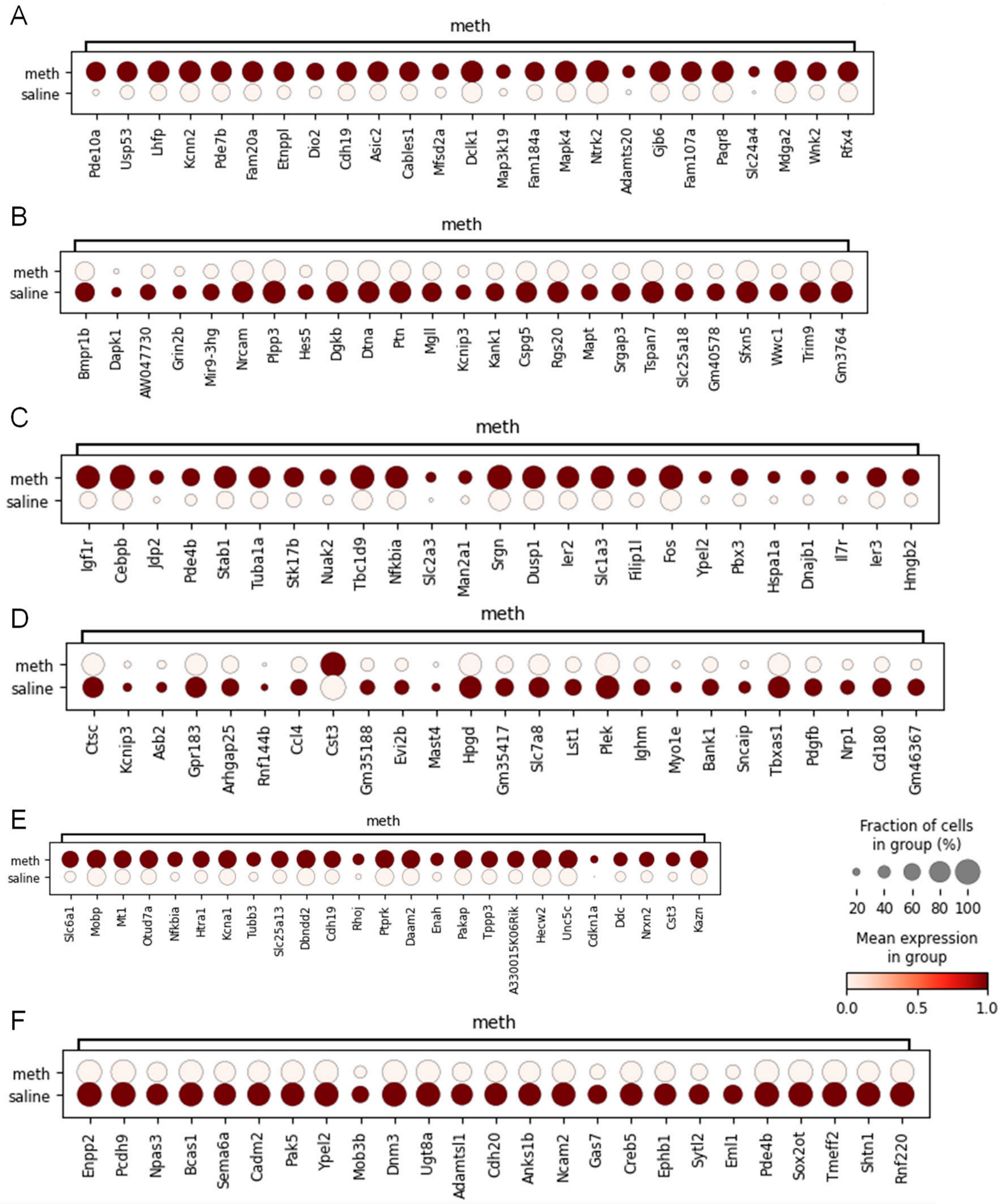

The top 25 (A) upregulated and (B) downregulated DEGs in astrocytes clusters in saline- and Meth-administered wildtype mice. The top 25 (C) upregulated and (D) downregulated DEGs in microglia clusters in saline- and Meth-administered wildtype mice. The top 25 (E) upregulated and (F) downregulated DEGs in oligodendrocytes clusters in saline- and Meth-administered wildtype mice.

Figure S3.

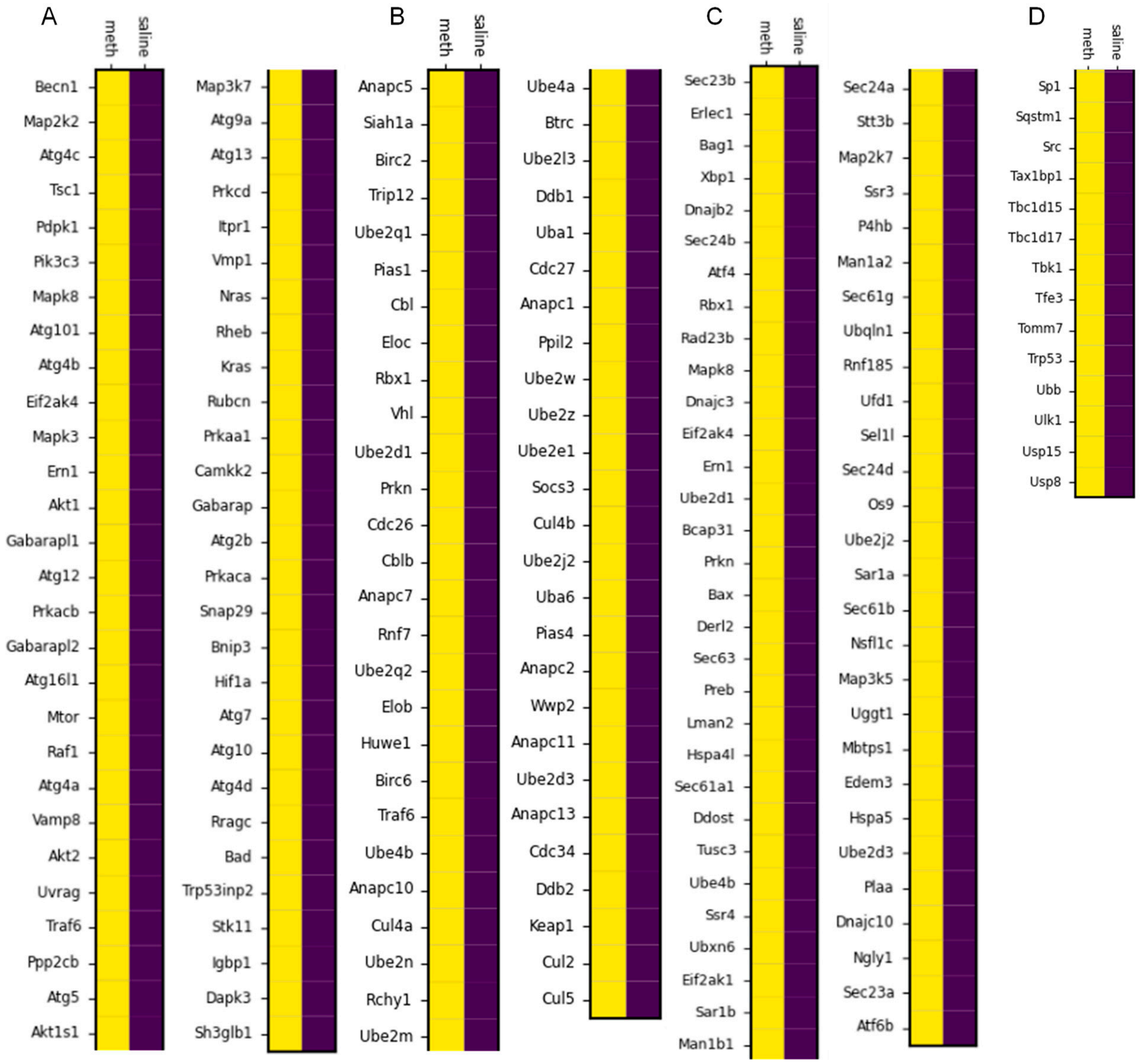

(A) Heatmap showing remaining DEGs in autophagy, (B) ubiquitin mediated proteolysis, (C) protein processing of endoplasmic reticulum, and mitophagy in saline- and Meth-administered groups.
